# Betanodavirus Induces Oxidative Stress-Mediated Cell Death That Prevented by Anti-Oxidants and Zfcatalase in Fish Cells

**DOI:** 10.1371/journal.pone.0025853

**Published:** 2011-10-03

**Authors:** Chih-Wei Chang, Yu-Chin Su, Guor-Mour Her, Chuian-Fu Ken, Jiann-Ruey Hong

**Affiliations:** 1 Laboratory of Molecular Virology and Biotechnology, Institute of Biotechnology, National Cheng Kung University, Tainan, Taiwan, Republic of China; 2 Institute of Bioscience and Biotechnology, National Taiwan Ocean University, Keelung, Taiwan, Republic of China; 3 The Department of Biotechnology, National Changhua University of Education, Changhua, Taiwan, Republic of China; University of Kansas Medical Center, United States of America

## Abstract

The role of oxidative stress in the pathogenesis of RNA nervous necrosis virus infection is still unknown. Red-spotted grouper nervous necrosis virus (RGNNV) induced free radical species (ROS) production at 12–24 h post-infection (pi; early replication stage) in fish GF-1 cells, and then at middle replication stage (24–48 h pi), this ROS signal may upregulate some expressions of the anti-oxidant enzymes Cu/Zn SOD and catalase, and eventually expression of the transcription factor Nrf2. Furthermore, both antioxidants diphenyliodonium and *N*-acetylcysteine or overexpression of zebrafish catalase in GF-1 cells also reduced ROS production and protected cells for enhancing host survival rate due to RGNNV infection.

Furthermore, localization of ROS production using esterase activity and Mitotracker staining assays found that the ROS generated can affect mitochondrial morphology changes and causes ΔΨ loss, both of which can be reversed by antioxidant treatment. Taken together, our data suggest that RGNNV induced oxidative stress response for playing dual role that can initiate the host oxidative stress defense system to upregulate expression of antioxidant enzymes and induces cell death via disrupting the mitochondrial morphology and inducing ΔΨ loss, which can be reversed by anti-oxidants and zfcatalase, which provide new insight into betanodavirus-induced ROS-mediated pathogenesis.

## Introduction

The Nodaviridae family of viruses contains two genera: beta-nodaviruses, which predominantly infect fish, and alpha-nodaviruses, which mostly infect insects [1]–[5]. Beta-nodaviruses are the causative agents for viral nervous necrosis (VNN), an infectious neuropathological condition characterized by necrosis of the central nervous system, including the brain and retina. Clinical signs include abnormal swimming behavior and darkening of the fish [2]. VNN can cause massive dying off the larvae and juvenile populations of several marine teleost species [6], and the disease manifestations of these viruses may correlate with modulation of innate or acquired immunity [4], [7]. Furthermore, beta-nodaviruses may prove useful as a model for understanding RNA virus-mediated pathogenesis and disease.

The nodavirus genome is comprised of two single-stranded molecules of positive polarity (RNA1 and RNA2) approximately 3.1 and 1.4 kb in length, respectively, and lacking a 3′ poly (A) extension [4]. RNA1 encodes an ≈110-kDa nonstructural protein designated RNA-dependent RNA polymerase (RdRp) or protein A. This protein is vital for replication of the viral genome. RNA2 encodes a 42-kDa capsid protein [4], [8], which may induce post-apoptotic necrotic cell death through a cytochrome *c* release-dependent pathway [9].

Alpha and beta-nodaviruses synthesize a sub-genomic RNA3 from the 3′ terminus of RNA1 during RNA replication, which encodes two small proteins, B1 and B2 [1], [10], [11]. In RGNNV, *B1* was identified as a novel anti-necrotic cell death gene; however, the precise death mechanism it influences remains unresolved [12]. B2 acts either as a host siRNA silencing suppressor in alpha- [7], [13], [14] and beta-nodavirus [10], or as an inducer of necrotic cell death in fish cells [11].

Oxidative stress has been implicated in the pathogenesis of various neurodegenerative diseases such as Alzheimer's disease and Parkinson's disease [15]–[16]. Oxidative stress occurs in cells when production of reactive oxygen species (ROS) exceeds the cell's endogenous antioxidant defenses [17]. In cells, major defenses against ROS include superoxide dismutases (SODs) and catalase [18]–[19]. SODs catalyze the dismutation of superoxide (O_2_^−^) to hydrogen peroxide (H_2_O_2_) and molecular oxygen (O_2_), and are located in the cytoplasm (Cu/Zn SOD) and mitochondria (Mn SOD) [20]–[21]. Catalase is a tetrameric iron porphyrin protein located in the peroxisome that further scavenges H_2_O_2_ to make H_2_O and O_2_ [22]. Expression of catalase and Cu/Zn SOD is constitutive, whereas expression of Mn SOD within the mitochondria is oxidant-stress induced [23]–[24].

Many RNA viruses [25], DNA viruses [26], and retroviruses [27] can trigger oxidative stress and induce host cell death in infected cells. Betanodavirus induced ROS production and its connection to pathogenesis has not been well-studied. Such studies may provide important insight into treatment.

Previous studies of beta-nodavirus-induced host cell death by Chen et al [28] showed that the RGNNV TN1 strain induced apoptosis and post-apoptotic necrosis in a grouper liver cell line (GL-av). RGNNV infection of fish cells induced loss of membrane potential (Δ**Ψ**), which was blocked by the Δ**Ψ** inhibitor bongkrekic acid (BKA) [28] as well as the Bcl-2 family member protein zfBcl-xL [29]. In addition, the RGNNV genome encoded two viral death inducers, protein α and B2. Protein α (42 kDa) could activate caspase-3 [30], triggering mitochondrial-mediated cell death [9] that could be blocked by the Bcl-2 member zfBcl-xL. The second death inducer B2 (encoded by sub-genomic RNA3) acted via a Bax-mediated pathway [11]. Furthermore, a novel anti-necrotic death gene, *B1*, contributed to the regulation of cell death at an early stage of replication [12]. In the present study, we found that RGNNV can induce ROS production, causing mitochondrial fragmentation that culminates in host cell death. We showed that the antioxidants, *N*-acetylcysteine (NAC) and diphenyliodonium (DPI, Complex I inhibitor) both block ROS-mediated cell death. Furthermore, zebrafish antioxidant gene catalase overexpression can markedly reduce ROS production and enhance cell viability. Our data provide new insights into the ROS-mediated pathogenesis of RNA viruses and design of potentially therapeutic agents.

## Materials and Methods

### Cell line and virus

The grouper fin cell line, GF-1, was obtained from Dr. Chi (Institute of Zoology and Development of Life Science) Taipei, Taiwan, ROC). GF-1 cells were grown at 28°C in Leibovitz's L-15 medium (Gibco BRL, Gaithersburg, MD) supplemented with 5% fetal bovine serum and 25 µg/ml of gentamycin. Naturally-infected red grouper larvae were collected in 2002 in the Tainan prefecture and were the source of the RGNNV Tainan No. 1 (RGNNV TN1) used to infect GF-1 cells in this study. The virus was purified as described by Mori *et al.* [6] and was stored at −80°C until use. The vial titer was determined using the TCID_50_, assay, according to Dobos *et al.* [31].

### Assay of ROS production in intact cells

ROS was assayed in living cells using the Image-iT LIVE Green Reactive Oxygen Species Detection Kit (Molecular Probes, Eugene, OR). The assay depends on staining by carboxy-H_2_DCFDA (5-[and-6-]-carboxy-2′,7′-dichlorodihydrofluorescein diacetate), a reliable fluorogenic marker of ROS formation in live cells. GF-1 and pBudCE4.1-zebra fish (zf)catalase-producing cells (10^5^ cells/ml) were cultured to monolayer confluence in 60-mm diameter Petri dishes or in 6-wells-plate for 20 h, rinsed twice with PBS, pre-treated with either N-acetyl-L-cysteine (NAC; 1 mM) or Diphenyleneiodonium chloride (DPI; 30 nM) for 2 h, and infected with RGNNV (MOI = 1) for 0, 12, 24, 48, or 72 h at 28°C. At the end of each incubation time in 60-mm diameter Petri dishes, the culture medium was aspirated, and the cells were washed with PBS and either incubated in the dark for 30 min with 500 µl of working solution (25 µM carboxy-H_2_DCFDA in PBS). The samples were examined immediately under a fluorescence microscope with 100 W halogen for 0.5 second using the following band-pass filters: 488-nm excitation and 515-nm long-pass filter for detection of fluorescein. The percentage of 200 cells at each time point was determined in triplicate, with each point representing the mean of three independent experiments. Error bars represent the SEM. All data were analyzed using either a paired or unpaired Student's *t*-test, as appropriate. *P<0.01 indicated a statistically significant difference between mean values of the groups. On the other hand (for counting total fluorescent amount assay in microplate), at each time points (0, 12, 24, 48 and 72 h pi), the 6-wells-plate was incubated in the dark for 30 min with 350 µl of working solution (25 µM carboxy-H_2_DCFDA in PBS). Then, samples counted the total fluorescence in per sample with a fluorescence microplate reader by using the following band-pass filters: 488-nm excitation and 515-nm long-pass filter for detection of fluorescein.

### Hydrogen peroxide (H_2_O_2_) assay

Cellular hydrogen peroxide (H_2_O_2_) was assayed using the Amplex Red Hydrogen Peroxide/Peroxidase Assay Kit (Molecular Probes). GF-1 cells (10^5^ cells/ml) were cultured to monolayer confluence in a 60-mm diameter Petri dishes for 20 h, rinsed twice with PBS, pre-treated with NAC (1 mM) or DPI (30 nM) for 2 h, infected with RGNNV infection (MOI = 1) for 0, 12, 24, 48, or 72 h at 28°C, washed with PBS, and lysed in 0.1 ml of lysis buffer (50 mM Tris HCl, pH 7.4, 150 mM NaCl, 1 mM EDTA, 1% Triton X-100, 0.5 mM PMSF) with shaking on the shaker at 4°C for 30 min for well lysis, and centrifuged (13,000 rpm, 2 min, 4°C) to pellet insoluble materials. The samples (50 µl of supernatant) were mixed with working solution (100 µM Amplex Red and 0.2 U/mL horseradish peroxidase [HRP]) and incubated at room temperature for 30 min in the dark. Fluorescence was measured in a microplate reader with excitation at 530 nm and fluorescence emission detection at 590 nm. Background fluorescence of the without-H_2_O_2_ control was subtracted from each reading [32].

### Selection of zfcatalase-producing GF-1 cells

A zebrafish catalase (zfCatalase) was cloned and inserted into the expression vector pBudCE4.1 (so-called pBudCE4.1-zfCatalase) by Dr. Ken [33]. Vector-producing (pBudCE4.1; as a negative control) and pBudCE4.1-zfCatalase-producing cells were obtained by transfection of GF-1 cells, respectively, and selection with Zeocin (500 µg/ml). Transcription of the inserted sequences was driven by the immediate-early promoter of human cytomegalovirus in these vectors. Selection time (2–2.5 months) varied based on cell-dependent properties.

### Western blot analyses

GF-1 cells and pBudCE4.1-zfCatalase-producing GF-1 cells either treated with NAC (1 mM) or DPI (30 nM) (all 10^5^/ml) were cultured in 60-mm diameter Petri dishes for 20 h to monolayer confluence, rinsed twice with PBS, and infected with RGNNV infection (MOI = 1) for 0, 24, 48, or 72 h at 28°C. At the end of each incubation time, the culture medium was aspirated. The cells were washed with PBS and lysed in 0.3 ml of lysis buffer (10 mM Tris, 20% glycerol, 10 mM SDS, and 2% ß-mercaptoethanol, pH 6.8). An aliquot of the lysate was used for SDS-PAGE [34]. Blots were incubated with polyclonal antibodies to protein A, α (9), mouse Cu/Mn SOD, catalase, Nrf2, or actin (1∶1500; Upstate, Charlottesville, VA, USA), and then with peroxidase-labeled goat anti-rabbit conjugate (1∶7500; Amersham Biosciences, Piscataway, NJ). Binding was detected by chemiluminescence and the signals were captured on Kodak XAR-5 film (Eastman Kodak, Rochester, NY) [35]. The protein expression level amounts were quantified by Personal Densitometer (Molecular Dynamic).

### Annexin-V–FLUOS staining

To identify apoptotic cells, phosphatidylserine externalization on the outer leaflet of apoptotic cell membranes was analyzed using annexin-V–fluorescein (36). GF-1 cells (10^5^/ml) were cultured to monolayer confluence in 60-mm diameter Petri dishes for 20 h, rinsed twice with PBS, treated with antioxidants NAC (1 mM) or DPI (30 nM) for 2 h, and infected with RGNNV (MOI = 1) for 0, 24, 48, and 72 h. At each time point, cells were removed from culture, washed with PBS, incubated 10–15 min with 100 µl of a HEPES-based annexin-V–fluorescein solution (Boehringer-Mannheim, Mannheim, Germany), and evaluated by fluorescence microscopy (488 nm excitation; 515 nm emission) [36].

### Flow cytometric analysis

Analyses of stained RGNNV-infected and uninfected GF-1 cells treated with antioxidants NAC (1 mM) or DPI (30 nM) for 2 h, and infected with RGNNV (MOI = 1) for 48 h, then were performed on a FACS Vantage cell sorter (Becton-Dickinson, San Jose, CA, USA). Images of PI red fluorescence, a 650-nm long-pass filter bandpass filter (FACS Vantage; the higher PI fluorescence intensity allowing separation of altered cells, PI^+^, from intact cells, PI^−^). Each analysis was done on at least 10,000 cells gated in the region of the cells on the basis of light scatter properties. Fluorescence data were displayed on one or two major peak scales [30].

### Evaluation of mitochondrial membrane potential (ΔΨ)

GF-1 cells (untreated, treated with NAC [1 mM], or treated with DPI [30 nM]) were cultured and infected as above. The culture medium was discarded, and each dish was incubated (37°C, 15–20 min) after addition of 500 µL of diluted MitoCapture reagent (Mitochondria BioAssay™ Kit; BioVision, Mountain View, CA) [28] and then examined immediately under a fluorescence microscope using the following band-pass filters: 488-nm excitation and 515-nm long-pass filter for detection of fluorescein and 510-nm excitation and 590-nm long-pass filter for detection of rhodamine.

### Quantification of cell viability

Vector control-4 (pBudCE4.1) and zfCatalase-3 producing GF-1 cells (all 10^5^/ml) were cultured in 60 mm diameter Petri dishes for 20 h, treated with antioxidants NAC (1 mM) or DPI (30 nM) for 2 h, and infected with RGNNV (MOI = 1) at 28°C for 0, 24, 48, or 72 h. At each time point, sets of Petri dishes were washed with PBS and treated with 0.5 ml of 0.1% trypsin-EDTA (Gibco, Grand Island, NY) for 1–2 min. Cell viability was determined in triplicate using a trypan blue dye exclusion assay [37]. Each data point represents the mean viability of three independent experiments ± SEM. Data were analyzed using either a paired or unpaired Student's *t*-test, as appropriate. A value of *p*<0.05 was taken to represent a statistically significant difference between mean values of groups.

### Cell counts and statistical analyses

The percentage of MMP loss and annexin V-fluorescein positive cells was determined in each sample by counting 200 cells. Each result is expressed as the mean ± the standard error of the mean (SEM). Data were analyzed using either a paired or unpaired Student's *t*-test, as appropriate. A value of *p*<0.05 was taken to indicate a statistically significant difference between group mean values.

## Results

### RGNNV infection can induce ROS production in fish cells

**Figure 1:**
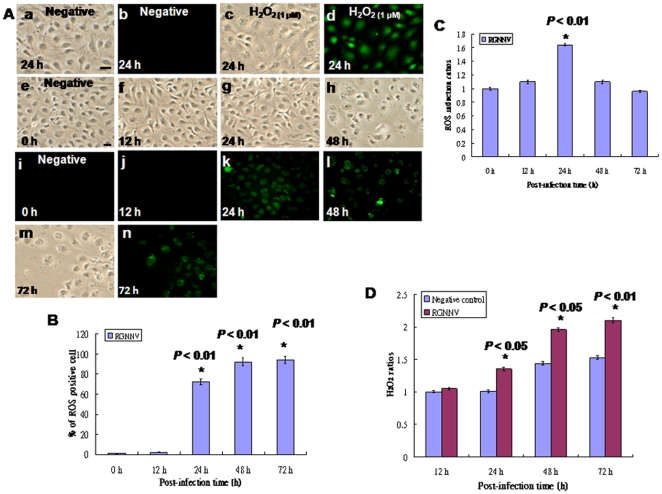
Identification of RGNNV infection induces ROS production in GF-1 cells. (A) ROS production (indicated by arrows) at 0, 12, 24, 48, and 72 h pi by cells infected with RGNNV (MOI = 1). The negative control (0 h) (see e and i); 24 h negative control (see a and b); RGNNV-infected groups at 12, 24, 48, and 72 h pi (see f–m and j–n, respectively; Green fluorescent cells as the ROS production positive cells); positive control H_2_O_2_ (1 µM) at 24 h post-incubation (see c and d; Green fluorescent cells as the ROS production positive cells). Scale bar = 10 µm. (B) The percentage of ROS-producing cells was counted at 0, 12, 24, 48, and 72 h, and shows a significant increase over time. In this and all subsequent figures (unless otherwise noted) data are presented as the percentage of 200 cells at each time point determined in triplicate, with each point representing the mean of three independent experiments. The vertical bars indicate ± the standard error of the mean (SEM). All data were analyzed using either a paired or unpaired Student's *t*-test, as appropriate. Statistically significant was defined at *P*<0.01. (C) The ratios of ROS-producing cells were counted by fluorescence microplate reader at 0, 12, 24, 48, and 72 h, and showed a significant increase at 24 h pi, but not at 48 h and 72 h pi because those times left few cells in wells. *P<0.01 indicated a statistically significant difference between mean values of the groups. (D) Concentration of peroxide in medium of RGNNV-infected cells producing H_2_O_2_ ratio at 12, 24, 48, and 72 h pi, respectively. Peroxide concentration at each time point was determined in triplicate. *P<0.05.

In previous studies, RGNNV induced mitochondria-mediated cell death, but whether this is connected to the induction of ROS production is unknown. In the present study, RGNNV infection induced ROS production at 24 h pi (Fig. 1A: g and k), 48 h pi (Fig. 1A: h and l), and 72 h pi (Fig. 1A: m and n) when compared to at 12 h pi (Fig. 1A: f and j) and the negative control (0 h; [Fig. 1A: e and i], 24 h; [Fig. 1A: a and b]) and positive control cells (H_2_O_2_ treatment (1 µM); Fig. 1A: c and d), which directly counted 200 cells under a microscope. The percentage of cells producing RGNNV-induced ROS increased very quickly from almost 0 at 0 h to 72 at 24 h pi, 92 at 48 h pi, and 94 at 72 h pi (Fig. 1B), which counted by per 200cells in three times independent experiments. Furthermore, we have counted the totally fluorescence in 6-wells with fluorescence microplate reader (Fig. 1C). The fluorescence ratio were mild increased at 12 h pi (1.1-folds) and apparently increased at 24 h pi (1.65-folds) as compared with 0 h (mock group, as 1-fold), but almost maintained base level at 48 h pi (1.1-folds) and was lower than base level at 72 h pi (0.8-folds) because this stage just left few cells (about 15–20%) in wells.

Furthermore, cellular hydrogen peroxide (H_2_O_2_) was detected by conversion to O_2_ using The Amplex RedHydrogen Peroxide/peroxidase Assay Kit. H_2_O_2_ ratio was increased in infected cells at 12 (1.1-folds), at 24 h (1.35-folds), at 48 h (1.95-folds), and at 72 h (2.15-folds) pi as compared with negative control cells at 12 h (1-fold, as a base level), at 24 h (1-fold), at 48 h (1.4-folds), and at 72 h (1.55-folds) (Fig. 1D), in agreement with ROS production results (Fig. 1A–B).

### RGNNV infection can upregulate anti-oxidant enzymes Cu/Zn SOD and catalase in GF-1 cells

**Figure 2 pone-0025853-g002:**
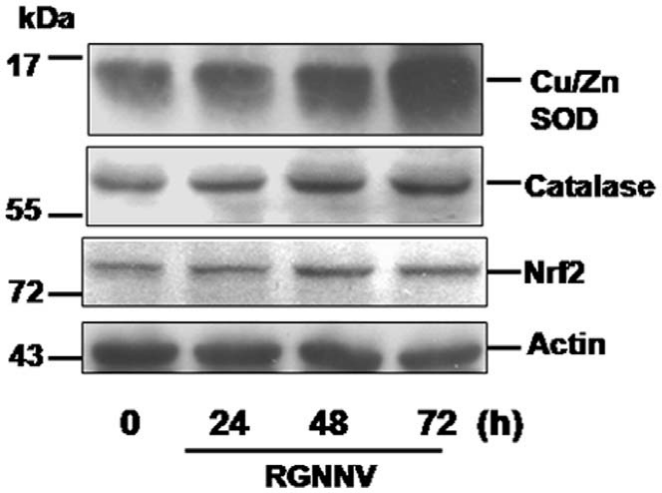
Western blot analysis of RGNNV infection up-regulates anti-oxidant enzymes Cu/Zn SOD and catalase or transcriptional factor Nrf2 in GF-1 cells at middle replication stage. The GF-1 cells pre-treated with antioxidants either NAC (1 mM) or DPI (30 mM) for two hours, then infected with RGNNV (MOI = 1) for different time incubations at 0, 24, 48, and 72 h pi. Samples were electrophoresed on a SDS-polyacrylamine gel and electro-blotted to a NC membrane. The NC membrane was stained with mouse monoclonal IgG antibodies directed against Cu/Zn SOD (15-kDa; Cayman), Catalase (57-kDa; Rockland), and Nrf2 (74-kDa) (Rockland). The chemiluminescent signal was imaged on XAR-5 film (Kodak) using a 5-min exposure. Lanes 1–4, 30 ul of virus-infected GF-1 cells and corresponded to 0, 24 h, 48 h and 72 h pi, respectively. The actin internal is also shown.

The oxidative stress-induced ROS production may induce generation of host anti-oxidant enzymes such as superoxide dismutase (SOD) or catalase. In our system, RGNNV infection either apparently upregulated the Cu/Zn SOD or mild upregulates catalase, and Nrf2 as shown by western blot analysis (compare Fig. 2, lanes 2–4; 24 h, 48 h, and 72 h pi, respectively, with Fig. 2, lane 1 [0 h]) that catalase, and Nrf2 gene expression level have checked by RT-PCR and received a similar results to western blot analysis at 48 h and 72 h pi.

### Anti-oxidant drugs DPI and NAC can reduce ROS production and inhibit RGNNV-induced cell death

**Figure 3 pone-0025853-g003:**
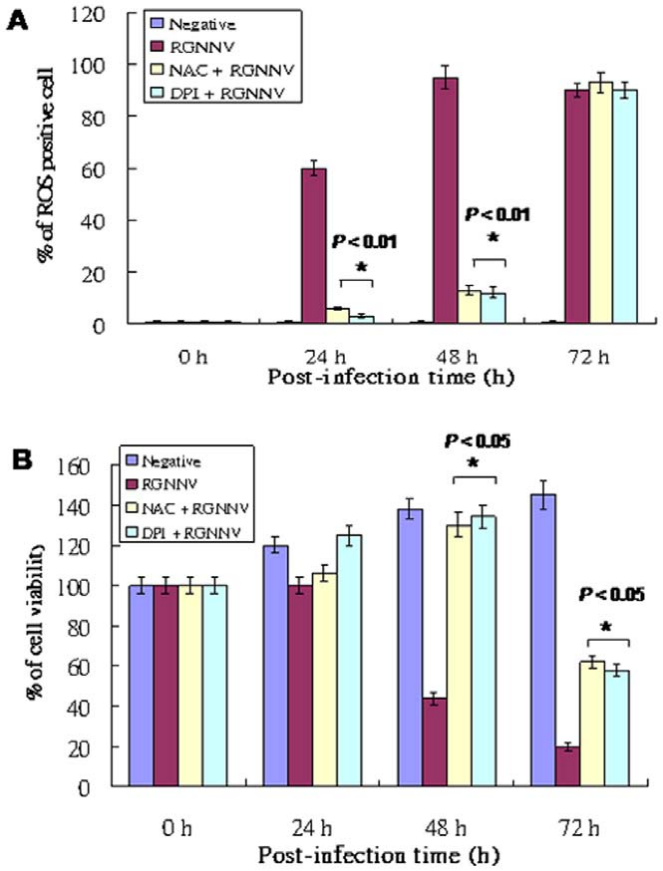
Influence of anti-oxidants NAC and DPI treatments on ROS production and cellular viability during RGNNV infection. (A) The GF-1 cells pre-treated with antioxidants either NAC or DPI for two hours, then infected with RGNNV (MOI = 1) for different time incubations. The number of ROS producing cells infected with RGNNV TN1 at 0, 24, 48, and 72 h pi was assayed with the Image-iT LIVE Green Reactive Oxygen Species Detection Kit. Data are the percentage of 200 cells at each time point, determined in triplicate, with each point representing the mean of three independent experiments; error bars represent the SEM. The data were analyzed using either a paired or unpaired Student's *t*-test. as appropriate. *P<0.01. (B) The viability of GF-1 cells infected with RGNNV and treated with or without NAC or DPI at 0, 24, 48, and 72 h pi in triplicate by using a trypan blue dye exclusion assay [37]. The data were analyzed using either a paired or unpaired Student's *t*-test. as appropriate. *P<0.05.

To determine whether blockade of ROS production can rescue cells from damage and death, the anti-oxidants NAC and DPI were tested. We found that antioxidants groups either NAC or DPI also can reduce ROS production apparently, at 24 h pi (55%, 57%), at 48 h pi (84.5%, 86.5%) as compared with RGNNV-infected groups 0 h (1%), 24 h (60%) and 48 h pi (96.5%) and negative control all 1% from 0 h to 72 h. On the other hand, at 72 h pi, antioxidants also shown no prevent effect that ROS positive cells up to 90%, which may lose their drugs activity in this time. Moreover, the viability assay (Fig. 3B) was determined in triplicate using a trypan blue dye exclusion assay [37]. We found that both drugs block cell death by approx 90% at 48 h pi and approx 38% at 72 h pi, compared to RGNNV-infected cells at 48 and 72 h pi. The cell number counted as a 100% at 0 h that some groups (at 24 h, 48 h and 72 h in negative controls) their viability were increased more than 100%.

**Figure 4 pone-0025853-g004:**
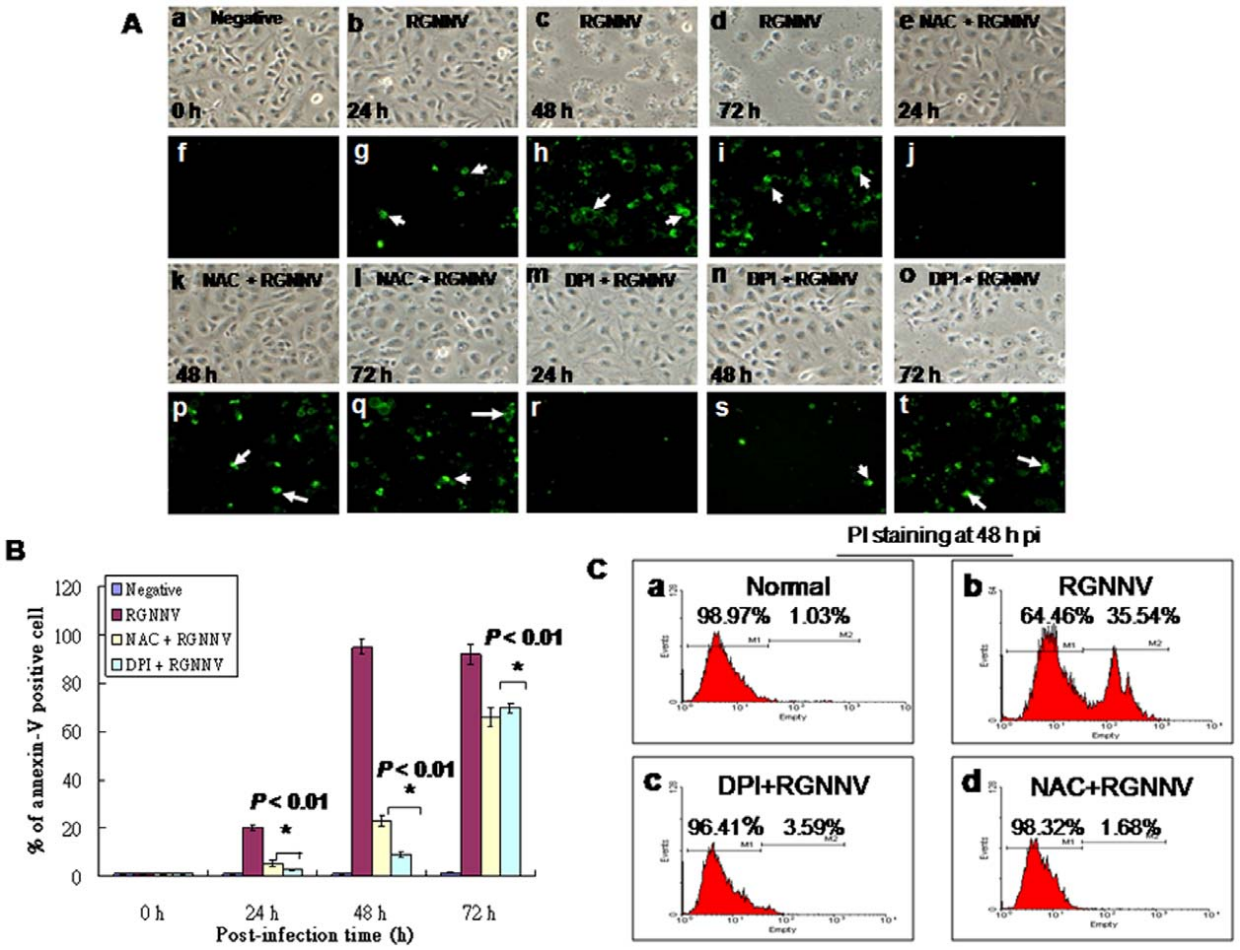
Identification of anti-oxidants treatment can reduce apoptotic/necrotic death of cells infected with RGNNV. (A) Phase-contrast and fluorescent micrographs of annexin-V–stained, RGNNV-infected GF-1 cells without drug-treatment at 0 h (a and f), 24 h (b and g), 48 h (c and h), and 72 h (d and i) or with NAC-treatment at 24 h (e and j), 48 h (k and p), and 72 h (l and q) and DPA-treatment at 24 h (m and r), 48 h (n and s), and 72 h (o and t). Annexin-V–positive cells (necrotic cells) are indicated by arrows. Scale bar = 20 µm. (B) The number of annexin-V–positive cells after infection with RGNNV at 0, 24, 48, and 72 h. Statistical comparisons were made using either a paired or unpaired Student's *t*-test, as appropriate. *P<0.05. (C) Examples of flow cytometric profiles in 48 h pi. RGNNV-infected cell and plus anti-oxidants treatment cells PI staining fluorescence was measured from 10,000 cells. Numbers in second peak scales (PI^+^) show late apoptotic/secondary necrotic cell percentages respectively. Viable cell percentage (PI^−^) is shown in first peak.

Furthermore, to detect the apoptotic cells in anti-oxidants treatment with annexin-V assay, we found that both NAC and DPI also shown very well prevention efficacy at 24 h (NAC, Fig. 4A: e and j; DPI, Fig. 4A: m and r; Annexin V-positive as indicated by arrows), 48 h (NAC, Fig. 4A: k and p; DPI, Fig. 4A: n and s; Annexin V-positive as indicated by arrows), 72 h (NAC, Fig. 4A: l and q; DPI, Fig. 4A: o and t; Annexin V-positive as indicated by arrows) pi when compared with RGNNV infection group at 24 h (Fig. 4A: b and g), 48 h (Fig. 4A: c and h), 72 h (Fig. 4A: d and i) pi and negative control 0 h (Fig. 4A: a and f). The percentages of annexin-V positive cells were reduced with NAC and DPI treatment (Fig. 4B) at 48 h pi by approx 70% and at 72 h pi by approx 20%. Compare with RGNNV-infected and negative control cells. On the other hand, apoptotic cells in percent of total number (PI^+^) (Fig. 4C) in RGNNV-infected cell at 48 h pi by approx 35.5% (Fig. 4C:b) as compare with normal control 1% (Fig. 4B:a), DPI treatment plus RGNNV-infected group 3.5% (Fig. 4C:c) and NAC plus RGNNV-infected group 1.6% (Fig. 4C:d), which reserved a consistent results that antioxidants can rescue cell death from RGNNV infection at middle replication stage (at 48 h pi), but did not at late replication stage (72 h pi), we supposed antioxidants NAC and DPI that may face the half-life problem.

### Zfcatalase overexpression can reduce RGNNV-induced ROS-mediated cell death

**Figure 5:**
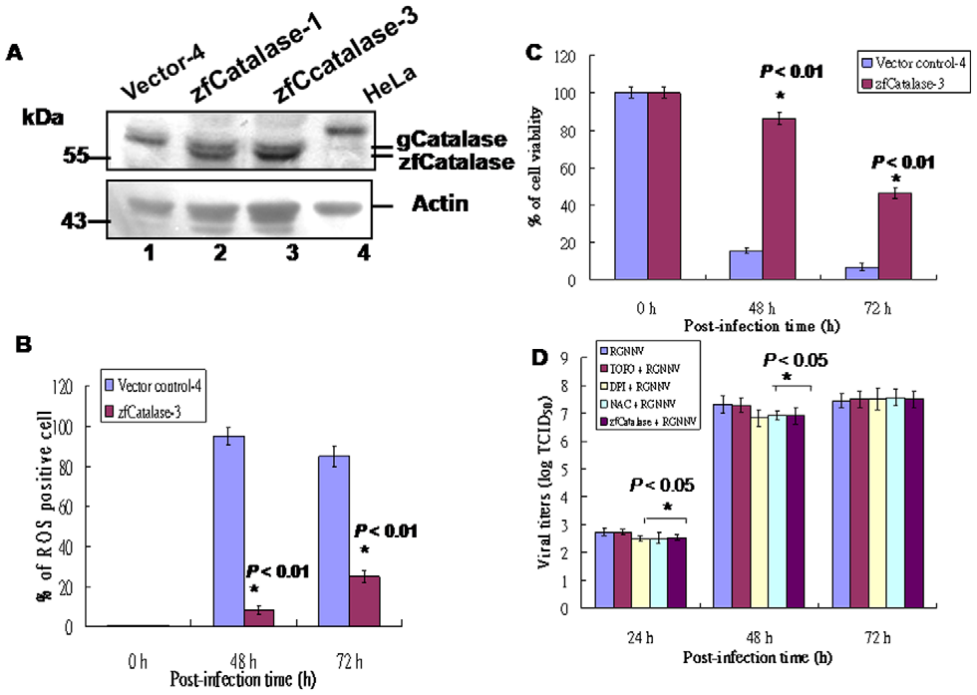
Identification of zebrafish catalase overexpression can reduce RGNNV-induced ROS-mediated cell death and viral titers in GF-1 cells. (A) Western blot analysis of zebrafish catalase-producing cell lines in GF-1 cells after selection with Zeocin (500 µg/ml). The stable clones are zfCatalase-1 (lane 2), zfCatalase-3 (lane 3), and vector control-4 (lane 1). HeLa cell lysate serves as a positive control (lane 4). Actin used as an internal loading control. (B) The number of ROS-producing cells after infection with RGNNV (MOI = 1) at 0, 48, and 72 h. Statistical comparisons were made using either a paired or unpaired Student's *t*-test, as appropriate. *P<0.01. (C) The viability of cells transfected with vector control-4 or zfcatalase-3 and infected with RGNNV was determined at 0, 48, and 72 h pi in triplicate by using a trypan blue dye exclusion assay [37]. Statistical comparisons were made using either a paired or unpaired Student's *t*-test, as appropriate. *P<0.01. (D) Viral titers were assays in GF-1 cell line by using at 48 h and 72 h pi samples. Statistical comparisons were made using either a paired or unpaired Student's *t*-test, as appropriate. *P<0.05.

Finally, to determine whether anti-oxidant enzymes can block ROS production and affect host cellular viability, zebrafish catalase-producing cells (zfcatalase-1 and -3) were selected. Western blot analysis (Fig. 5A, lanes 2–3) showed that GF-1-zfcatalase-3 cells may express more zfcatalase than do GF-1-zfcatalase-1 cells, so GF-1-zfcatalase-3 cells were chosen for further study. The number of ROS producing cells were reduced approx 80% (at 48 h pi) and 60% (at 72 h pi; Fig. 5B) by zfcatalase and viability was enhanced approx 70% (at 48 h pi) and 40% (at 72 h pi; Fig. 5C), which may have a more efficiency on cellular viability than antioxidants at 72 h pi.

Furthermore, we want to know whether ROS production can affect the viral replication. In viral titer assays (Fig. 5D), at early stage (24 h p.i.) and middle stage (48 h pi), all mild reduces about 0.3–0.5 log in zfcapalase-contained cell groups, NAC and DPI, but shown not significant different at 72 h pi (late replication stage), which received a consistent results in Figs. 3A–B and 5B–C that oxidative stress can mild regulate the viral replication at early and middle replication stages, but lose its regulatory function at late replication stage, which the viral titer reached a plateau within intact cell.

### RGNNV-induced ROS production related to affect in mitochondrial morphology and loss of delta psi (ΔΨm)

**Figure 6:**
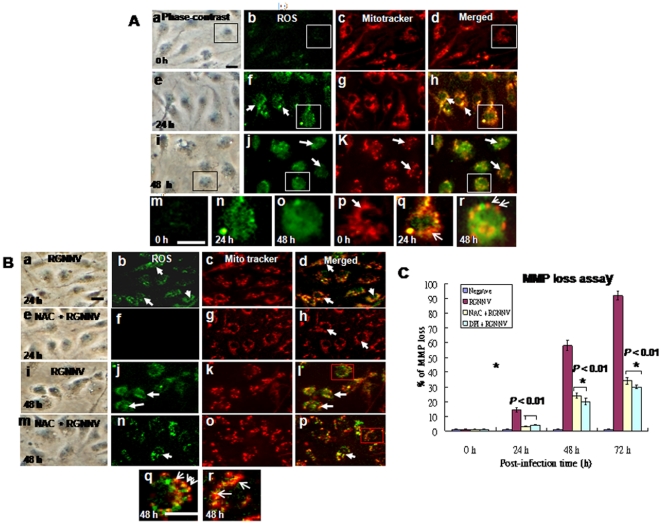
Identification of RGNNV-induced ROS production and the effect of ROS on mitochondrial morphology and loss of ΔΨ in GF-1 cells. Phase-contrast and fluorescence micrographs showing ROS production (the Image-iT LIVE Green Reactive Oxygen Species Detection Kit) and mitochondrial morphology (stained by Mito tracker) were in the same cells. (A) RGNNV-infected GF-1 cells at 0 h (a–d), 24 h (e–h; ROS produced in cells, and 48 h (i–l). The elongated mitochondrial network at 0 h in A:d is indicated by arrow in A:p. ROS production at 0 h in A:m is indicated in open square in A:b; at 24 h pi in A:n and p is indicated open square in A:f and h; at 48 h pi in A:o and r is indicated in open square in A:j and l. Breakdown of mitochondrial fission (indicated by arrows) at 48 h pi in A:r is indicated open square in A:l. Scale bar = 10 µm. (B) RGNNV-infected GF-1 cells treated with NAC at 24 h (e–h) and 48 h pi (m–p), or not treated at 24 h (a–d) and 48 h pi (i–l) with RGNNV infection. Blockade of mitochondrial breakdown in RGNNV-infected GF-1 cells at 48 h pi in B:p is indicated open square in B:l, which were appeared some dot of mitochondria and indicated by arrow; without RGNNV-infected cells at 48 h pi in B:r is indicated open square in B:p, which have shown more longer mitochondria in length that indicated by arrow. Scale bar = 10 µm. (C) The effect of anti-oxidants NAC and DPI on ΔΨ in cells infected with RGNNV. The Δ**Ψ** (MMP loss) of RGNNV-infected GF-1 cells treated or not treated with NAC or DPI was determined at 0, 24, 48, and 72 h pi in triplicate. Statistical comparisons were made using either a paired or unpaired Student's *t*-test, as appropriate. *P<0.05.

Furthermore, to determine whether ROS production can affect mitochondrial morphology, ROS production and mitochondrial morphology changes were monitored in RGNNV-infected cells. RGNNV-induced ROS production in green fluorescence cells occurred mainly in ells at 24 h pi (compare Fig. 6A, e–h, n and q to Fig. 6A, a–d, m and p [negative control cells at 0 h]). By 48 h pi, ROS had been localized in the cytoplasm (Fig. 6A, i–l, o and r), which may induce breakdown down of mitochondria (Fig. 6A, k and l) with MitoTracker staining in red fluorescence cells (A:k) and merged cells (A:l; orange fluorescence cells). Compare the enlarged image of Fig. 6A, r (inset of Fig. 6A, l at 48 h) with that of Fig. 6A, p (inset of Fig. 6A, d) that apparently breakdown down of mitochondria indicated by arrows.

NAC treatment blocked ROS production at 24 h pi (compare Fig. 6B, e–h with Fig. 6B, a–d, [RGNNV-infected cells]) and reduced ROS production in cells at 48 h pi (compare Fig. 6B, m–p with Fig. 6B, i–l [RGNNV-infected cells]). Compare the enlarged image of Fig. 6B, r (inset of Fig. 6B, p at 48 h) with that of Fig. 6B, q (inset of Fig. 6B, l) that apparently prevent breakdown down of mitochondria in length by NAC treatment, which indicated by arrows.

In addition, it is not known whether inhibition of the RGNNV-induced-ROS production can block **ΔΨ** loss. Mitochondrial function was evaluated using MitoCapture Reagent (Apoptosis Detection, Mitochondria BioAssay™ Kit). The Mitocapture dye aggregates in the mitochondria of healthy cells and fluoresces red. In apoptotic cells, the dye cannot accumulate in mitochondria, remains as monomers in the cytoplasm, and fluoresces green. Furthermore, in **ΔΨ** loss rate, counts of cells with loss of **ΔΨ** at 0, 24, 48, and 72 h pi (Fig. 6C) that both drugs blocked **ΔΨ** loss up to 34% at 48 h pi and 58% at 72 h pi, respectively.

## Discussion

Betanodavirus causes viral nervous necrosis (VNN) and the infected fish to lie on its side, float belly up, or swim abnormally. Histopathological changes include extensive cellular vacuolation and necrotic neuronal degeneration in the central nervous system and retina [38]. The molecular cell death mechanisms involved in the pathogenesis of this disease are still unknown. The present study demonstrated a novel ROS-mediated cell death pathway, i.e., death via mitochondria-produced oxidative stress, which may impact the host anti-oxidant enzyme system and mitochondria-mediated cell death. Characterization of processes underlying beta-nodavirus ROS-mediated cell death may help clarify viral molecular pathogenesis mechanisms and therapeutic drug development.

### Virus-induced ROS production (12–24 h pi; early replication stage) regulates the expression of anti-oxidant enzymes and transcription factor involved in maintaining pro-/antioxidant balance in the middle replication stage (24–48 h pi)

ROS are implicated in a wide variety of pathologies, including malignant diseases, type II diabetes, atherosclerosis, chronic inflammatory processes, ischemia/reperfusion injury, and several neurodegenerative diseases [39]. Reactive oxygen species (ROS) have been attributed potential dangerous molecules as they can oxidize lipids and DNA and limit the availability of NO. In recent years that ROS are important second messengers that several sources of ROS, such as mitochondria, xanthine oxidase, NO synthase and cytochrome P450 monooxygeneases have all been shown to be of relevance ROS production [40]. Complex I and complex III of the electron-transport chain are the major sites for ROS production [41], [42]. Complex I inhibition by rotenone can increase ROS generation in submitochondrial particles [41], [43]. The oxidation of either complex I or complex II substrates in the presence of complex III inhibition with antimycin A increases ROS [43], [44]. On the other hand, ROS can play a regulatory role in cellular metabolic processes by activation of various enzymatic cascades as well as transcriptional factors to upregulate expression of anti-oxidant enzymes such as superoxide dismutase and glutathione peroxidase [39]. In our system, RGNNV induced ROS production (Fig. 1) apparently at 24 h pi and then mild upregulated the catalase and transcription factor Nrf2, which is a cellular sensor of chemical- and radiation-induced oxidative and electrophilic stress [45] and controls the expression and coordinated induction of a battery of defensive genes encoding detoxifying enzymes and antioxidant proteins. However, it is not known whether Nrf2 upregulated the anti-oxidant enzymes in our system. On the other hand, RGNNV infection did apparently upregulate Nrf2, Cu/Zn SOD and catalase at 48 h pi (Fig. 2), which may help to restore ROS homeostasis. Furthermore, anti-oxidants NAC and DPI (Figs. 3 and 4) and overexpression of zfcatalase (Fig. 5) did inhibit RGNNV-induced ROS production and induction of cell death, eventually enhancing host cell viability [39], but in late replication stage (72 h pi) did not reduced ROS production in Fig. 3A that antioxidants may be gradually lost those activity.

In addition to NADPH oxidases (Noxs), recently received most attention. The family of NADPH oxidases of seven members, Nox1–Nox5 and Doux1 and Doux2 [41] are all producing ROS. Interesting different types of ROS are produced by NADPH oxidases. Nox4 predomainantly generates hydrogen peroxide (H_2_O_2_), whereas superoxide anions (O_2_^−^) are produced by Nox1 and Nox2. Recently, In HCV system, these induced a persistent elevation of Nox1 and Nox4 and increased nuclear localization of Nox4 in hepatocytes in vitro and in the human liver that Nox protein are likely to act as a persistent, endogenous source of ROS during HCV-induced pathogenesis [46]. In our system, we have checked the Nox4 expression level with RGNNV infection. In the result of RGNNV infection did not increase the Nox4 protein level (data not shown), which may meant complex I and complex III are more predominately to produce ROS in this fish cells with RGNNV infection.

### Whether ROS molecules can regulate viral replication?

In recent years that ROS are important second messengers that ROS whether can regulate the viral replication is still few to known, eventually in HIV [27] and HCV [46] systems.

In our system, in viral titers assay, we interestingly found that ROS stress response in middle replication stage (at 48 h pi) can reduce about 0.2–0.3 log in antioxidant (DPI and NAC) or antioxidant enzyme (zfCatalase) (Fig. 5D). But in late replication stage (at 72 h pi), did not shown the prevent ability in viral titer reducing that may be antioxidants [47] activity gradually loss or antioxidant enzyme zfCatalase might be shout-off in this stage, but are still verified. Taken together of Figs. 1–5 results, we found that RGNNV-induced ROS signal may mild modulate the viral replication in early and middle replication stage (24–48 h pi), after that we proposed that ROS balance was severely lost because some viral death inducers [9], [11], [48] may apparently express and caused ROS production at late replication stage. At early and middle replication stages, ROS-mediated response may play dual role either enhance the viral replication or modulate the oxidative stress response for upregulation of antioxidant enzymes such as catalase or Cu/Zn SOD, but how to regulate the viral replication is still unknown.

### RGNNV-induced oxidative stress disrupts mitochondrial function and affects the mitochondrial morphology at middle replication stage (at 24–48 h pi)

Mitochondria are dynamic organelles that can change in number and morphology in healthy cells [49]. The mitochondria provide a myriad of services to the cell, including energy production, calcium buffering, and regulation of apoptosis [50]. Mitochondria also play a key role in modulation of Ca^2+^ homeostasis and oxidative stress [51] and form a network that can effectively deliver energy or channel calcium between different areas of the cell [52]. The number and morphology of mitochondria are precisely controlled through mitochondrial mitochondria-shaping proteins [53]–[54], including both fusion members (e.g., large GTPase mitofusins, Mfn1 and Mfn2 [55], and optic atrophy protein a (Opa1) [56] and fission members such (e.g., Fis1 [57] and dynamin-related protein 1 (Drp1) [58]. Equilibrium between mitochondrial fusion and fission controls the morphology of the mitochondria. Disruption of fusion fragments the normal tubular network of mitochondria into rods or spheres; disruption of fission generates elongated, interconnected tubules that often cluster perinuclearly [54]. Mitochondrial fission accompanies several types of apoptotic cell death and appears to be associated with progression along the apoptotic pathway [59].

Complex I and complex III of the electron-transport chain are the major sites for ROS production [41]–[42]. Complex I inhibition by rotenone can increase ROS generation in submitochondrial particles [42]–[43]. The oxidation of either complex I or complex II substrates in the presence of complex III inhibition with antimycin A increases ROS [43]–[44].

In that the current study, RGNNV infection induced ROS production during early replication at 24 h pi (Fig. 6A: e–h, n and q), and ROS localization was mainly in cytoplasm and mitochondria (Fig. 6A: i–l, o and r) at 48 h pi. In addition, ROS production disrupted mitochondrial morphology (Fig. 6A: k, l) converting the normal tubular network of mitochondria into fragments (rods or spheres: Fig. 6A: r) or interconnected tubules that often cluster perinuclearly through middle replication stage at 48 h pi. Antioxidant NAC treatment blocked the ROS production in the cytoplasm and mitochondria (compare Fig. 6B: e–h with Fig. 6B: a–d [RGNNV-infected cells, no treatment]) but reducing mitochondrial fragmentation in length (Fig. 6B: m–p) that especially in enlarged image Fig. 6B:r as compared with no NAC treatment Fig. 6B:q at 48 h pi. These results suggest the involvement of ROS production as well as other factors in the induction of mitochondrial fragmentation. Finally, NAC and DPI (Fig. 6C) also can blocked RGNNV-induced MMP loss up to 30% (48 h pi) and 60% (72 h pi; late replication stage) during replication, which support RGNNV induction of ROS affect GF-1 viability.

**Figure 7:**
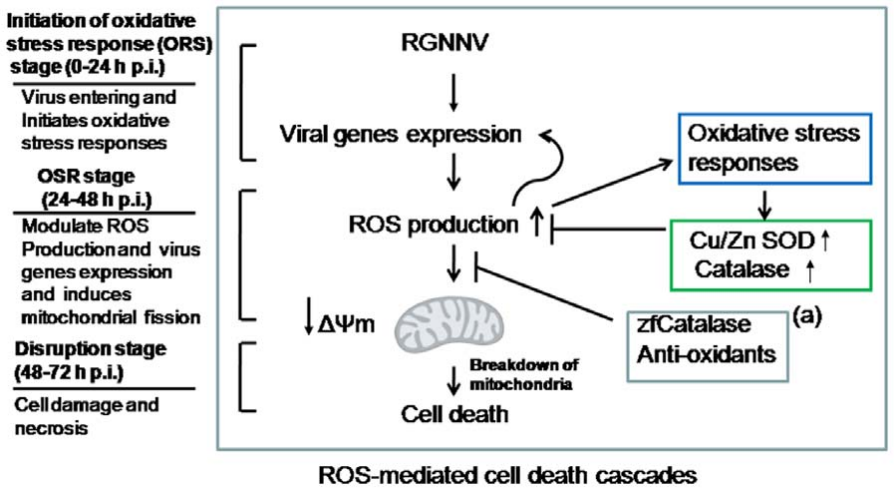
A schematic illustrating our hypothesis of RGNNV infection induced ROS-mediated cell death. RGNNV first can enter and express viral early gene at early replication stage [0–24 h pi; initiation of oxidative stress response (OSR) stage], then induces ROS production apparently in mitochondria and initiates oxidative stress responses. Furthermore, at middle replication stage (24–48 h pi; OSR stage), oxidative stress responses are dramatically (1) up-regulating the antioxidants enzymes Cu/Zn SOD and catalase, (2) affecting the viral replication for mild increasing virus titer and (3) eventually producing mitochondrial breakdown as a fission action. Finally, cells undergo a ROS-mitochondria-mediated cell death at late replication stage (at 48 h-72 h pi; disruption stage). RGNNV-induced death signaling is halted by anti-oxidants DPI and NAC or anti-oxidant enzyme zfCatalase over-expression (a) for reducing ROS production and enhancing cell viability.

In summary (Fig. 7), beta-nodavirus enters the host cell where viral genome replication and viral gene expression occur during the early stages of replication (0–24 h pi). Then, this viral expression produces reactive oxygen species in cells and then initiates an oxidative stress response (OSR). Furthermore, at middle replication stage (24–48 h pi), this ROS oxidative stress response stage further ROS up-regulates the transcription factor Nrf-2 or anti-oxidant enzymes Cu/Zn SOD and catalase to maintain intracellular ROS equilibrium [60] and may modulate viral replication for reducing virus titer. On the other hand, antioxidants NAC and DPI and anti-oxidant enzyme zfcatalase also blocked mitochondria-mediated ROS production and reducing consequently cell death. If reduction in oxidative stress is insufficient (late replication stage; 48–72 h pi), cell death via the caspase-independent pathway [61] and disruption in the late of replication stage (48–72 h pi) may occur. Therefore, our study provides new insights into a possible mechanism of RGNNV-induced pathogenesis and points to potential targets for therapy directed at the source of ROS.
